# A cross sectional survey on health-related quality of life of elementary school students using the Korean version of the EQ-5D-Y

**DOI:** 10.7717/peerj.3115

**Published:** 2017-03-21

**Authors:** Sang-Kyu Kim, Min-Woo Jo, Seon-Ha Kim

**Affiliations:** 1 Department of Preventive Medicine, Dongguk University College of Medicine, Gyeongju-si, South Korea; 2 Department of Preventive Medicine, University of Ulsan College of Medicine, Seoul, South Korea; 3 Department of Nursing, Dankook University College of Nursing, Cheonan, South Korea

**Keywords:** Health-related quality of life, EQ-5D-Y, Children, Korea

## Abstract

**Background and Objective:**

The Korean version of the EQ-5D-Y was launched in 2015 by the EuroQol group. Currently, there is no HRQOL data obtained by using the EQ-5D-Y in Korea. This study aimed to measure health-related quality of life of Korean elementary school students using the EQ-5D-Y.

**Methods:**

Elementary school students were recruited from 11 primary schools in Gyungbuk, South Korea. The EQ-5D-Y was self-administered in the sample population. Demographic characteristics were collected from the subjects’ parents or guardians. The percentage of respondents reporting problems and VAS scores were calculated. Feasibility of the EQ-5D-Y was assessed by analysing the proportion of missing responses. The percentage of reported problems on the dimensions and VAS score between groups were compared by demographic factors.

**Results:**

A total of 2,494 questionnaires were collected. There were 24 (0.96%) missing responses on the EQ-5D-Y and 187 (7.5%) missing VAS score responses. The proportion of reported problems ranged from 2.3% on the mobility dimension to 9.8% on the “having pain or discomfort” dimension. There was no significant difference in the proportion of problems by age group in male participants. However, in females, the older group reported significantly more problems on the “having pain or discomfort” and “feeling worried, sad, or unhappy” dimensions compared to the younger group. Students living with parents with the lowest educational level reported significantly more problems on the “looking after myself” and “doing usual activities” dimensions than did those living with parents with higher levels of education.

**Discussion:**

This study showed the distribution of health-related quality of life and explored the feasibility of the EQ-5D-Y for measuring health-related quality of life in Korean elementary school students. Further studies are required to examine other psychometric properties of the Korean EQ-5D-Y.

## Introduction

Interest in health-related quality of life (HRQOL) as an outcome measure in children and adolescents has increased in recent decades in both clinical and community-based research (Higginson & Carr, 2001; Solans et al., 2008). HRQOL is a significant outcome of care; however, it is argued that proxy estimation of children’s HRQOL (e.g., by parents or other caregivers) may not grant an accurate judgement (Sung et al., 2004; Prosser, Hammitt & Keren, 2007; Ungar, 2011). Accordingly, a number of instruments have been developed as generic, disease-specific, or preference-based instruments to assess HRQOL in children and adolescents (Rajmil et al., 2012; Solans et al., 2008; Chen & Ratcliffe, 2015). Generic instruments can be applied to a wide variety of populations. They allow for broad comparisons of the relative impact of various health care interventions or comparisons of HRQOL between healthy and ill children (Fayers & Machin, 2000). Disease-specific instruments can be more sensitive at detecting clinically important differences or changes in specific disease groups (Wiebe et al., 2003). However, they cannot measure the HRQOL of patients without the specific disease (e.g., cancer), and it is not possible to compare the HRQOL of patients with other diseases (e.g., diabetes mellitus) or of a healthy population. Preference-based instruments are general instruments with a prespecified algorithm that assigns a single number, called a preference weight (where 1 represents a state of perfect health and 0 represents death) for each health state defined by the instrument (Chen et al., 2015). Calculated preference weight can be used for cost-utility analysis in both clinical and public health settings.

EQ-5D-3L is one of the most popular preference-based generic instruments (Rasanen et al., 2006). It was originally developed for people aged 16 and older (EuroQol Group, 2014), although several studies used the EQ-5D-3L index to assess HRQOL in children with diseases (Kuhlmann et al., 2016; Matza, Secnik & Mannix, 2005; Protudjer et al., 2015). The EuroQoL Group developed the EQ-5D-Y, a child-friendly version of the EQ-5D-3L, to be used in children and adolescents (Wille et al., 2010). The EQ-5D-Y is comprised of the same five dimensions with three levels as is the EQ-5D-3L, but its wording was tailored to the target population of 8- to 18-year-olds (Wille et al., 2010). The feasibility of the EQ-5D-Y is supported by absence of missing or ambiguous answers and a high self-completion rate (Burström, Svartengren & Egmar, 2011; Jelsma, 2010; Ravens-Sieberer et al., 2016; Scalone et al., 2011; Burström et al., 2014; Bergfors et al., 2015; Robles et al., 2015). Jelsma (2010) reported that the EQ-5D-Y performed better than did the standard EQ-5D. The convergent and divergent validity of the EQ-5D-Y tested with a child-specific instrument was satisfactory (Eidt-Koch, Mittendorf & Greiner, 2009; Ravens-Sieberer et al., 2016; Scalone et al., 2011; Burström et al., 2014; Bergfors et al., 2015). Known-group construct validity of the EQ-5D-Y was supported (Burström, Svartengren & Egmar, 2011; Ravens-Sieberer et al., 2016; Scalone et al., 2011; Burström et al., 2014; Robles et al., 2015), and a healthy group reported health problems (response level two or higher on the EQ-5D-Y) less frequently than did an unhealthy group. Older groups tended to report more problems than younger groups in all dimensions of the EQ-5D-Y except ‘looking after myself,’ (Burström, Svartengren & Egmar, 2011; Bergfors et al., 2015). The HRQOL of children tended to be positively associated with parents’ educational level (Wu et al., 2014). The test–retest reliability of the EQ-5D-Y has been demonstrated in different populations, including children and adolescents with diseases (Ravens-Sieberer et al., 2016; Scalone et al., 2011; Robles et al., 2015).

The EQ-5D-Y Korean version was launched in 2015 by the EuroQol group. Currently, there is no published HRQOL data obtained using the EQ-5D-Y instrument in Korea. This study aimed to assess HRQOL in Korean elementary school students using the EQ-5D-Y, to compare HRQOL by demographic factors, and to explore the feasibility of using the Korean EQ-5D-Y to assess HRQOL. Such initial data may prove useful when comparing the HRQOL of a variety of Korean elementary school students in future studies.

## Materials & Methods

### EQ-5D-Y

The EQ-5D-Y consists of the descriptive system of the EQ-5D-Y and the EQ visual analogue scale (EQ VAS). It asks a child to self-rate his or her health on the present day. Its descriptive system comprises the same five dimensions as does the EQ-5D-3L, except that it uses child-friendly wording (mobility, looking after myself, doing usual activities, having pain or discomfort, and feeling worried, sad or unhappy). The five dimensions have different underlying constructs. Each dimension has three levels: ‘no’, ‘some’, and ‘a lot of’ problems in four of the dimensions, and ‘not’, ‘a bit’, and ‘very’ in the last dimension. The intervals between the three levels are not necessarily equal. The EQ VAS records the respondent’s self-rated health (“How good is your health TODAY?”) on a vertically numbered, visual analogue scale from 0 to 100. Its endpoints are labelled ‘The worst health you can imagine’ at 0 and ‘The best health you can imagine’ at 100 (EuroQol Group, 2014). A unique health state is defined by recording the problem level from each of the 5 dimensions using the EQ-5D-Y. Each state is referred to in terms of a 5-digit code. For example, the 11111 state represents a full health state with no problems in any dimension, whereas 11312 represents no problems with mobility or looking after oneself, a lot of problems with usual activities, no pain or discomfort, and a bit worried, sad or unhappy. Thus, a total of 243 (5^3^) possible health states can be defined with this instrument. The use of the EQ-5D-Y Korean version in this study was approved by the EuroQoL group.

### Procedure

This study is a cross-sectional survey. This study was approved by the Institutional Review Board of Dongkook University (110757-201605-HR-06-03). Elementary school students were recruited by convenience sampling from 11 primary schools in the area around Gyungbuk, South Korea. All participating schools enrolled normally developing, healthy children. Questionnaires were sent home with children in sealed envelopes, with an informative newsletter and consent form for their parents or guardians. One copy of the written consent form was signed by both the parents or guardians and the child. The questionnaires consisted of the EQ-5D-Y and questions on demographic information. The student was informed that the EQ-5D-Y was to be self-completed. Demographic characteristics, including age, gender, grade, and parents’ educational level, were obtained from their parents or guardians. No reminders to reply were sent. The survey was conducted between May 10 and July 1, 2015. Of 2,731 questionnaires, 2,494 were completed (91.3%). The response rate ranged from 71.8 to 100% across schools.

### Analysis

The feasibility of using the EQ-5D-Y descriptive system and EQ VAS to measure the health-related quality of life of Korean elementary school students was assessed by analysing the proportion of missing or invalid values on the EQ-5D-Y descriptive system and EQ VAS. Differences in demographic characteristics (age, gender, and grade) between valid responders and responders who missed questions were assessed using the Mann–Whitney U test or Fisher’s exact test. The frequency and percentage of reported problems and the VAS scores of participants were calculated. The reported EQ-5D-Y health profiles were also explored. The difference in the percentage of respondents reporting problems by age, gender, and the parent’s or guardian’s education level was determined with a Chi-square test. Differences in EQ VAS score between groups were determined with an independent *t*-test or analysis of variance (ANOVA). All statistical analyses were performed using SAS software version 9.1 (SAS institute, Cary, NC, USA).

## Results

Of the 2,494 responses, 24 (0.96%) had missing data for at least one dimension of the EQ-5D-Y. There were 16, 15, 16, 16, and 21 missing responses for the 5 dimensions, respectively. A total of 187 (7.5%) subjects did not include responses for EQ VAS. There was no invalid answer on the EQ-5D-Y descriptive system or EQ VAS. The range of age of missing responders was from seven to 13 years old. There was no significant difference in demographic characteristics (i.e., age, gender, and grade) between valid responders and missing responders. Responses with missing values for the EQ-5D-Y descriptive system (24 cases) and participants who were six years old (two cases) and 13 years old (2 cases) were excluded from the analysis. Therefore, 2,466 cases were used for the final analysis.

The age of participants ranged from seven to 12 years, with an average age of 9.5 years (SD, 1.7). Males comprised 51.4% of the participants. Grade in school was evenly distributed. Almost half of students in each grade were male, and the gender distribution by grade was not statistically different (*p* value = 0.892, data not shown). Approximately half of parents graduated from university (Table 1).

**Table 1:** Characteristics of the study subjects (*N* = 2,466).

Variables		*n* (%)	
Age (years), Mean (SD)		9.53 (1.7)
Gender	Male	1,255 (51.4)
Grade	1	403 (16.3)
	2	420 (17.0)
	3	394 (16.0)
	4	407 (16.5)
	5	408 (16.6)
	6	434 (17.6)
Parent education level	Middle school or less	104 (4.4)
	High school	1,082 (45.7)
	College or more	1,181 (49.9)

**Notes.**

Number of missing values in gender: 22.

Number of missing values in parents’ educational level: 99.

The proportions of reported problems by dimension and EQ VAS score are summarized in Table 2. The proportion of reported problems in the ‘having pain or discomfort’ dimension was highest (9.9%), followed by that in the ‘feeling worried, sad, or unhappy’ dimension (9.3%). High ceiling effects were seen in all dimensions. The mean VAS score was 89.7 (SD, 12.4). A total of 49 distinct EQ-5D-Y health states were reported. The most frequent EQ-5D-Y health state was ‘11111’ (i.e., full health), at 81.2%, followed by the ‘11112’ and ‘11121’ health states (Table 2).

**Table 2:** Percentage of reported problems, VAS score, and EQ-5D-Y health profiles in the study sample.

		*n* (%)
Mobility (walking about)		
No problems		2,409 (97.7)
Some problems		52 (2.1)
A lot of problems		5 (0.2)
Looking after myself		
No problems		2,408 (97.7)
Some problems		51 (2.1)
A lot of problems		7 (0.3)
Doing usual activities		
No problems		2,370 (96.1)
Some problems		91 (3.7)
A lot of problems		5 (0.2)
Having pain or discomfort		
No problems		2,224 (90.2)
Some problems		233 (9.5)
A lot of problems		9 (0.4)
Feeling worried, sad or unhappy		
No problems		2,236 (90.7)
Some problems		222 (9.0)
A lot of problems		8 (0.3)
VAS, mean(SD)		89.7 (12.4)
VAS, median(range)		90 (17–100)
Frequently reported EQ-5D-Y Health state profiles	11111	2,003 (81.2)
	11112	127 (5.2)
	11121	127 (5.2)
	11122	45 (1.8)
	11211	21 (0.9)
	12111	16 (0.7)
	11212	13 (0.5)
	21121	13 (0.5)
	21111	10 (0.4)

Comparisons of reported problems and VAS scores on the EQ-5D-Y by age and gender are shown in Table 3. There was no significant difference amongst male participants by age group. However, in females, the older group reported significantly more problems on the ‘having pain or discomfort’ and ‘feeling worried, sad or unhappy’ dimensions compared with the younger group. The younger group showed significantly higher EQ VAS scores than did the older group in both genders. Males (regardless of age) reported significantly more problems in the ‘looking after myself’ and ‘doing usual activities’ dimensions than did females (*p*-value = 0.034, and <0.001, respectively, data not shown). There were no significant differences in other dimensions between gender groups. Older groups (regardless of gender) reported more problems than did younger groups only in the ‘having pain or discomfort’ dimension (*p*-value = 0.0062, data not shown).

**Table 3:** Comparison of reported problems and VAS score on the EQ-5D-Y by age and gender.

	Male				Female			
	7–9 years (*n* = 612) *n* (%)	10–12 years (*n* = 643) *n* (%)	χ^2^ or *t*	*p*-value^*^	7–9 years (*n* = 589) *n* (%)	10–12 years (*n* = 600) *n* (%)	χ^2^ or *t*	*p*-value^*^
Mobility (walking about)								
No problems	602 (98.4)	625 (97.2)	1.952	0.162	578 (98.1)	583 (97.2)	1.206	0.272
Any problems^†^	10 (1.6)	18 (2.8)			11 (1.9)	17 (2.8)		
Looking after myself								
No problems	591 (96.6)	628 (97.7)	1.358	0.244	579 (98.3)	591 (98.5)	0.074	0.786
Any problems^†^	21 (3.4)	15 (2.3)			10 (1.7)	9 (1.5)		
Doing usual activities								
No problems	575 (94.0)	613 (95.3)	1.182	0.277	578 (98.1)	585 (97.5)	0.556	0.456
Any problems^†^	37 (6.1)	30 (4.7)			11 (1.9)	15 (2.5)		
Having pain or discomfort								
No problems	552 (90.2)	570 (88.7)	0.794	0.373	552 (93.7)	533 (88.8)	8.886	0.003
Any problems^†^	60 (9.8)	73 (11.4)			37 (6.3)	67 (11.2)		
Feeling worried, sad or unhappy								
No problems	557 (91.0)	587 (91.3)	0.030	0.863	541 (91.9)	531 (88.5)	3.761	0.053
Any problems^†^	55 (9.0)	56 (8.7)			48 (1.9)	69 (11.5)		
VAS, mean (SD)	91.4 (11.0)	88.6 (12.5)	3.970	<.0001	91.9 (10.6)	87.2 (14.4)	6.210	<.0001

**Notes.**

* *p*-value by Chi-square test or student *t*-test.

† Levels 2 and 3 were collapsed.

The results of reported problems and EQ VAS scores by parental educational level are shown in Table 4. Students living with parents with the lowest educational level reported significantly more problems in the ‘looking after myself’ and ‘doing usual activities’ dimensions than did those living with more educated parents. EQ VAS scores by parental educational levels were not significantly different from each other.

**Table 4:** Comparison of reported problems and VAS scores in the EQ-5D-Y by parents’ educational level.

	Middle school or less^a^ (*n* = 104) *n* (%)	High school^b^ (*n* = 1,082) *n* (%)	College or more^c^ (*n* = 1,181) *n* (%)	*X*^2^ or F	*p*-value^*^	Post hoc grouping
Mobility (walking about)						
No problems	100 (96.2)	1,060 (98.0)	1,153 (97.6)	1.484	0.476	
Any problems^†^	4 (3.9)	22 (2.0)	28 (2.4)			
Looking after myself						
No problems	95 (91.4)	1,054 (97.4)	1,161 (98.3)	19.978	<.0001	a < b, c
Any problems^†^	9 (8.7)	28 (2.6)	20 (1.7)			
Doing usual activities						
No problems	95 (91.4)	1,043 (96.4)	1,139 (96.4)	7.004	0.030	a < b, c
Any problems^†^	9 (8.7)	39 (3.6)	42 (3.6)			
Having pain or discomfort^*^						
No problems	93 (89.4)	980 (90.6)	1,061 (89.8)	0.409	0.815	
Any problems^†^	11 (10.6)	102 (9.4)	120 (10.2)			
Feeling worried, sad or unhappy						
No problems	90 (86.5)	984 (90.9)	1,071 (90.7)	2.177	0.337	
Any problems^†^	14 (13.5)	98 (9.1)	110 (9.3)			
VAS, mean(SD)	89.4 (11.8)	89.6 (12.5)	90.1 (12.2)	0.480	0.617	

**Notes.**

* *p*-value by Chi-square test or ANOVA.

† Levels 2 and 3 were collapsed.

## Discussion

This was the first study that assessed HRQOL using the EQ-5D-Y in Korea. Our study was based on convenience sampling and had a large sample size.

The number of missing responses in the EQ-5D-Y dimensions and the VAS in this study were low, similar to those reported in previous studies (Ravens-Sieberer et al., 2016; Wille et al., 2010). A multinational study by Ravens-Sieberer et al. (2016) reported that the proportion of missing responses in the EQ-5D-Y dimensions and VAS are 0 to 2% and 0 to 9%, respectively, depending on country. Students are more likely to find it difficult to assign a direct VAS score than to choose an option on the EQ-5D-Y. The high completion rate in this study may be the result of some respondents completing the EQ-5D-Y with assistance at home, although we informed the students in writing that they should complete the questionnaires by themselves. Jelsma (2010) noted more missing responses in the lower grades and reported that only the youngest group (mean age of 14) showed missing responses. Although our study subjects were younger than those in Jelsma’s study, the proportion of missing responses was lower and the age of missing responders varied from 6 to 13 years old. Overall, the low number of missing or inappropriate responses supports the feasibility of using the Korean EQ-5D-Y to assess HRQOL.

Similar to surveys using the EQ-5D-3L in the general population, a considerable ceiling effect was observed in this study. This is because of the nature of the EQ-5D-Y having only three levels per dimension and is partly due to our subjects representing the general population. Burström et al. (2014) compared HRQOL in children and adolescents between the general population and those being treated for health conditions. Participants with functional disabilities reported more problems with a much lower ceiling effect on the EQ-5D-Y (Burström et al., 2014; Domellöf, Hedlund & Ödman, 2014). Ravens-Sieberer et al. (2016) previously indicated that the EQ-5D-Y might not be very useful for discriminating between respondents in the general population. Therefore, further studies regarding the discriminatory ability of the EQ-5D-Y for assessment of HRQOL are required that use study subjects with a wider age range and different problems.

The trends of reported problems in the five dimensions in this study were similar to the findings from other countries, including low problem rates in the ‘mobility’, ‘looking after myself’, and ‘doing usual activity’ dimensions but relatively high problem rates in the ‘having pain or discomfort’ and ‘feeling worried, sad or unhappy’ dimensions (Burström et al., 2014; Ravens-Sieberer et al., 2016; Scalone et al., 2011; Wille et al., 2010). Nevertheless, in this study, the proportion of individuals reporting problems was much lower (9.3%) in the ‘having pain or discomfort’ and ‘feeling worried, sad or unhappy’ dimensions than in Germany (37.3% and 39.6%, respectively), Italy (39.0% and 39.0%, respectively), Spain (20.0% and 23.1%, respectively) (Ravens-Sieberer et al., 2016), Sweden (32.4% and 15.1%, respectively) (Burström, Svartengren & Egmar, 2011), South Africa (47.3% and 38.7%, respectively) (Jelsma, 2010), Canada (45.5% and 36.1%, respectively) (Wu et al., 2014), and Australia (37.4% and 45.8%, respectively) (Chen et al., 2015). Children had a lower rate of reporting problems in all dimensions of the EQ-5D in Korea compared to other countries, and they showed similar trends as did adults in Korea (Kim et al., 2013). Asians are more likely to report a higher ceiling effect than are non-Asians, although their objective health and disease conditions are equal, and this difference is thought to be associated with racial/ethnic differences (Fu & Kattan, 2006).

The mean VAS score in this study was 89.7 (SD, 12.4), which is similar to that in previous studies on the general population (ranging from 77.3 in South Africa Wille et al., 2010 to 89.1 in Sweden Burström, Svartengren & Egmar, 2011). Nine EQ-5D-Y health states were reported in this study and 81.2% of respondents reported the 11111 state. In studies on EQ-5D-Y health states in the general population in Sweden, 63.4% of respondents were in full health (Burström et al., 2014) and 48.9% of patients with asthma reported the full health state (Bergfors et al., 2015). There may be cultural differences in the ways students report their problems. In the author’s comparison of age-standardized problem reporting in adults in the general population, Korean people commonly showed lower problem rates on all EQ-5D-3L dimensions compared with respondents in 10 European countries.

In a comparison of EQ-5D-Y responses by gender, the male group reported significantly more problems in the ‘looking after myself’ and ‘doing usual activities’ dimensions than did the female group. The reason for this phenomenon is uncertain and more research is needed.

In a comparison of EQ-5D-Y responses by age, older groups reported more problems than did younger groups in the ‘having pain or discomfort’ dimension. Similarly, in a study by Burström et al. (2014), girls reported more problems only in the dimension of ‘feeling worried, sad, or unhappy’ than did boys, and older individuals tended to report more problems than did younger individuals in the ‘having pain or discomfort’ and ‘feeling worried, sad or unhappy’ dimensions. Burström, Svartengren & Egmar (2011) showed 8-year-olds reported significantly more problems than did 12-year-olds in the dimension ‘looking after myself’ and ‘feeling worried and sad’. These two studies conducted in Sweden for general public, however, there are differences in the report rate of the problem by age. School-age children develop their ability to adapt and exercise through their relationships with school and people around them. Further research is needed on how this affects the quality of life.

We also explored the differences in HRQOL according to parental education level. The HRQOL of participants living with parents with the lowest educational level was lower than that of other groups in the ‘looking after myself’ and ‘doing usual activities’ dimensions of the EQ-5D-Y. Wu et al. (2014) reported a positive association between parental education and the VAS-based index using the EQ-5D-Y. Burström, Svartengren & Egmar (2011) also reported a positive association between the highest parental education level and VAS score, although the association was not statistically significant.

There are several limitations of this study. First, the EuroQol group recommended that the EQ-5D-Y be used for children aged 8–18. However, some of our subjects were seven years old. This might be not problematic because the EQ-5D-Y is simple and easy to administer. In fact, the rate of missing data in this age group was not higher than that in other age groups. Juniper et al. (1997) investigated the minimum age and reading level necessary to competently follow HRQOL instruments in children with asthma. They found that all children from seven to 17 years of age provided very reliable data on the Paediatric Asthma Quality of Life Questionnaire and the Health Utility Index, and that the Feeling Thermometer required children to have at least grade two (age 7–8 years) reading abilities (Juniper et al., 1997). Canaway & Frew (2013) also reported that children aged 6–7 years can feasibly complete utility instruments when surveys are administered by an interviewer. The target population of the EQ-5D-Y could be expanded through further research in younger populations. Second, our study could not evaluate the validity and reliability of the EQ-5D-Y due to limited tools to determine convergent validity and the study design without retest. For example, we did not investigate morbidity, and thus cannot compare HRQOL between a healthy group and an unhealthy group. Third, we did not ensure that the children completed the EQ-5D-Y themselves at home. Although we provided written instructions, parents or guardians may have filled out the EQ-5D-Y. There have been no previous publications regarding the psychometric properties of the Korean EQ-5D-Y, although those of the EQ-5D-Y have been examined in other countries. Further research on the validity and test-retest reliability of the Korean EQ-5D-Y and research into HRQOL in paediatric patients are required.

## Conclusion

This study demonstrated the distribution and feasibility of the EQ-5D-Y for assessing HRQOL in Korean elementary school students for the first time. However, further studies are required to examine the validity, test–retest reliability, and sensitivity to change of the Korean EQ-5D-Y for elementary school students with a wider range of clinical conditions.

##  Supplemental Information

10.7717/peerj.3115/supp-1 Data S1 Raw data

## Additional Information and Declarations

### Data Availability

The following information was supplied regarding data availability: The raw data has been supplied as Data S1.
